# Racial Differences in Insular Connectivity and Thickness and Related Cognitive Impairment in Hypertension

**DOI:** 10.3389/fnagi.2017.00177

**Published:** 2017-05-31

**Authors:** Ganesh B. Chand, Junjie Wu, Deqiang Qiu, Ihab Hajjar

**Affiliations:** ^1^Division of General Internal Medicine and Geriatrics, Department of Medicine, Emory University School of Medicine, AtlantaGA, United States; ^2^Department of Radiology and Imaging Sciences, Emory University School of Medicine, AtlantaGA, United States; ^3^Department of Biomedical Engineering, Georgia Institute of Technology, Emory University, AtlantaGA, United States; ^4^Department of Neurology, Emory Alzheimer’s Disease Research Center, Emory University School of Medicine, AtlantaGA, United States

**Keywords:** cognitive impairment, cognitive racial disparity, salience network, default-mode network, central-executive network, Granger causality, cortical thickness, functional magnetic resonance imaging (fMRI)

## Abstract

Hypertensive African–Americans have a greater risk of cognitive impairment than hypertensive Caucasian–Americans. The neural basis of this increased risk is yet unknown. Neuroimaging investigations suggest that the normal neural activity comprises complex interactions between brain networks. Recent studies consistently demonstrate that the insula, part of the salience network, provides modulation effects (information flow) over the default-mode and central-executive networks in cognitively normal subjects, and argue that the modulation effect is declined in cognitive impairment. The purpose of this study is to examine the information flow at the nodes of three networks using resting state functional magnetic resonance imaging (MRI) data in cognitively impaired hypertensive individuals with the African–Americans and the Caucasian–Americans races, and to compare the thickness of impaired node between two racial groups. Granger causality methodology was used to calculate information flow between networks using resting state functional MRI data, and FreeSurfer was used to measure cortical thickness from T1-weighted structural images. We found that negative information flow of the insula in both African–Americans and Caucasian–Americans, which was in contrast with previously reported positive information flow in this region of normal individuals. Also, significantly greater negative information flow in insula was found in African–Americans than Caucasian–Americans (Wilcoxon rank sum; *Z* = 2.06; *p* < 0.05). Significantly, lower insula thickness was found in African–Americans compared with Caucasian–Americans (median = 2.797 mm vs. 2.897 mm) (Wilcoxon rank sum; *Z* = 2.09; *p* < 0.05). Finally, the insula thickness correlated with the global cognitive testing measured by Montreal cognitive assessment (Spearman’s correlation; *r* = 0.30; *p* < 0.05). These findings suggest that the insula is a potential biomarker for the racial disparity in cognitive impairment of hypertensive individuals.

## Introduction

It is estimated that above 30% of population and 65% of older population have hypertension worldwide (Novak and Hajjar, 2010). Previous studies suggest that hypertensive individuals have a greater chance of occurring the dementia and physical disability than normotensive individuals (Johnson et al., 2008; Elias et al., 2012; Faraco and Iadecola, 2013). Cognitive functions, especially the executive functions (Chand and Dhamala, 2016b), are widely reported of being impaired in hypertensive individuals (Vicario et al., 2005; Gorelick and Nyenhuis, 2012; Hajjar et al., 2016). Individuals with hypertension have higher chances of occurring executive dysfunction earlier than individuals with normotension, which indicates a potential vascular-cognitive association (Oveisgharna and Hachinski, 2010). However, there are very limited neuroimaging studies that investigated the neural bases of hypertension in cognitive decline (Li et al., 2015). Former non-neuroimaging case studies further report that the African–Americans bear the greater burden of hypertension in the United States and have earlier onset of hypertension and larger hypertension-associated cognitive symptomatology and mortality than the other racial groups, including the Caucasian–Americans (Redmond et al., 2011; Hajjar et al., 2016). The neural mechanisms underlying this racial disparity is largely under-investigated so far. A triple brain network model—a model consisting of the brain’s default-mode, salience, and central-executive networks and their interactions—has been recently employed in the neuroimaging field to elucidate the differences in the connectivity patterns associated with the different levels of cognitive impairments such as higher versus lower impairment (Menon, 2011; Uddin, 2015). We therefore use this model to investigate whether there is a difference in the impairment at the nodes of triple network between the African–Americans and the Caucasian–Americans hypertensive cognitively impaired individuals.

Resting state functional MRI (rsfMRI) has been used to investigate the functional brain areas or neurocognitive networks (Biswal et al., 1995; Raichle, 2015). Recent neuroimaging investigations suggest that the neural basis of cognitive activity is related to a dynamically modulating interaction between multiple networks, including the salience, default-mode, and central-executive networks (Bressler and Menon, 2010; Menon, 2015; Uddin, 2015; Chand and Dhamala, 2016a). The key nodes of the default-mode network include the posterior cingulate and the ventromedial prefrontal cortices, the salience network encompasses the insula and the dorsal anterior cingulate cortices, and the central-executive network comprises the posterior parietal and the dorsolateral prefrontal cortices (Chen et al., 2013). It has been demonstrated that the insula and dorsal anterior cingulate of salience network are anatomically connected (Bonnelle et al., 2012; Jilka et al., 2014) and consist of a special type of neurons named von Economo neurons that relay information processed within these regions to other brain regions, including the nodes of default-mode and central-executive networks (Allman et al., 2005, 2010; Watson et al., 2006; Sridharan et al., 2008). This control signal by the insula and the dorsal anterior cingulate cortex has been suggested to be crucial for cognitive maintenance, including a rest, in cognitively normal individuals (Sridharan et al., 2008; Goulden et al., 2014; Chand and Dhamala, 2016a). Alternation in insula connectivity has been consistently implicated in diseases, including autism, frontotemporal dementia, and schizophrenia (Menon, 2011; Uddin, 2015), but it has not been elucidated in cognitively impaired hypertensive patients. Literature suggests that the insula and anterior cingulate cortex—the key regions of salience network—respond as the racially biased brain regions (Cao et al., 2015), such as the greater activity of insula to faces of foreign races than faces of the same race of the subject (Lieberman et al., 2005; Liu et al., 2015). However, the difference in information flow—a measure from information theory that can be quantified using Granger Causality analysis (Dhamala et al., 2008b; Chand and Dhamala, 2017)—in these regions between the African–Americans and the Caucasian–Americans themselves has not been previously investigated. Previous investigations consistently report that the functional changes of brain regions (or networks) are associated with the underlying structural changes of those regions (or networks) with the progression of diseases (Xie et al., 2012; Menon, 2015). Specifically, recent studies suggest that the insula thickness decreases with the progression of cognitive decline in mild cognitive impairment (MCI) patients (Hartikainen et al., 2012; Moretti, 2015). However, whether there is a difference in insula thickness of the cognitively impaired hypertensive patients between the African–Americans and the Caucasian–Americans races has not been reported.

Here, we seek to examine the difference in information flow using Granger causality (Dhamala et al., 2008b; Chand and Dhamala, 2017) at the default-mode, salience and central-executive nodes between the African–Americans and the Caucasian–Americans hypertensive cognitive impaired individuals. As the African–Americans have higher hypertension-associated cognitive symptomatology and mortality than the other racial groups (Redmond et al., 2011; Hajjar et al., 2016), we *hypothesized* that (1) the control signal of the insula of salience network over the default-mode and central-executive nodes is more impaired (more negative value) in the African–Americans than in the Caucasian–Americans. We further seek to examine the structural difference that could substrate this racial disparity by comparing the insula thickness between the two racial groups. To test this, we further *hypothesized* that (2) the insula thickness is lower in the African–Americans than in the Caucasian–Americans, and finally (3) lower insula thickness is associated with poorer cognitive performance.

## Materials and Methods

### Participants

This study was carried out in accordance with the recommendations of “Institutional Review Board (IRB) of Emory University” with written informed consent from all subjects. All subjects gave written informed consent in accordance with the Declaration of Helsinki. The study and the protocol were reviewed and approved by Institutional Review Board of Emory University. The informed written consent was provided by the participants before data collection. We recorded magnetic resonance imaging (MRI) data from 78 individuals who had hypertension and MCI. The inclusion criteria were: (a) age ≥ 55 years, (b) hypertension defined by systolic blood pressure ≥ 140 mm Hg or diastolic blood pressure ≥ 90 mm Hg and (c) MCI was assessed based on previously defined Diagnostic and Statistical Manual of Mental Disorders (DSM) criteria (Chao et al., 2009; Pa et al., 2009): Montreal cognitive assessment (MoCA) ≤ 26, clinical dementia rating score of 0.5, minimal functional limitation as reflected by the functional assessment questionnaire ≤ 7, and cognitive performance at the 10th percentile or below till the 2nd percentile on at least one of four screening tests—trail marking test B (Reitan, 1958; Tombaugh, 2004), Stroop interference (Stroop, 1935; Kimble et al., 2009), digit span forward and digit span backward (Humstone, 1919; Pa et al., 2009), verbal fluency and abstraction (Henley, 1969; Troyer et al., 1997). Trail marking test B screens the participant’s executive ability to draw a line from a ‘number’ to a ‘letter’ in ascending order such as ‘1’ to ‘A’, ‘A’ to ‘2’, ‘2’ to ‘B’, and so on. Strop interference effect measures the interference of predominant response in the reaction time of a task such as when the name of a word (say ‘red’) is printed in a different color (say ‘blue’), it takes longer time to name the color of that word compared to when the word (‘red’) matches the name of color (‘red’). Digit span forward and digit span backward test consists of two parts: first, the participant listens to and repeats a sequence of numbers, and second, the participant listens to a sequence of numbers and repeats those numbers in reverse order. The former part screens the short-term auditory memory while the latter part screens the participant’s ability to manipulate the verbal information based on the auditory information. The verbal fluency screens the participant’s fluency such as the participant is asked to tell as many words as the participant can that begin with certain letter (say ‘A’) in a limited time and the abstraction test screens the participant’s ability to deal with ideas such as how an orange and a banana are alike. These tests have been commonly used to screen the MCI patients (Chao et al., 2009; Pa et al., 2009). The participants exclusion criteria were: (a) systolic blood pressure > 200 mm Hg or diastolic blood pressure > 110 mm Hg, (b) renal disease or hyperkalemia, (c) active medical or psychiatric problems, (d) uncontrolled congestive heart failure (shortness of breath at rest or evidence of pulmonary edema on exam), (e) history of stroke in the past 3 years, (f) ineligibility for MRI (metal implants or cardiac pacemaker), (g) inability to complete cognitive test and MRI scan, (h) women of childbearing potential and (i) diagnosis of dementia (self-reported or care-giver reported). In total sample, the mean age was 66.9 years (*SD*: 9.7), 55.1% were women, mean education was 14.9 years (*SD*: 2.6), mean systolic blood pressure 144.4 mm of Hg (*SD*: 22.6), mean diastolic blood pressure 86.4 mm of Hg (*SD*: 12.8), and MoCA ranged from 11 (minimum value) to 26 (maximum value) with mean score of 21.9 (*SD*: 3.1). Out of 78 participants, there were 50 African–Americans and 28 Caucasian–Americans. In the African–Americans, the mean age was 66.3 years (*SD*: 8.9), 60% were women, mean education was 14.9 years (*SD*: 2.5), mean systolic blood pressure 141.8 mm of Hg (*SD*: 21.3), mean diastolic blood pressure 85.4 mm of Hg (*SD*: 12.0), and mean MoCA score was 21.2 (*SD*: 3.2). In the Caucasian–Americans, the mean age was 68.5 years (*SD*: 10.7), 46.4% were women, mean education was 15.0 years (*SD*: 2.9), mean systolic blood pressure 147.6 mm of Hg (*SD*: 24.0), mean diastolic blood pressure 87.0 mm of Hg (*SD*: 12.7), and mean MoCA score was 23.4 (*SD*: 2.4). The age, sex, education year, systolic blood pressure, and diastolic blood pressure were not statistically significant different between the African–Americans and the Caucasian–Americans, but the MoCA score was significantly lower in the African–Americans compared to the Caucasian–Americans (*p* < 0.003) as shown in **Table 1**.

**Table 1:** Mean scores (standard deviations) and statistical comparison between the African–Americans (AA) and the Caucasian–Americans (CA) regarding their age, sex, education, blood pressure (BP), and Montreal cognitive assessment (MoCA) score.

Characteristic *N*	Total sample 78	AA 50	CA 28	*p*-Value
Age, year	66.9 (9.7)	66.3 (8.9)	68.5 (10.7)	0.44
Sex, women	43 (55.1%)	30 (60%)	13 (46.4%)	0.25
Education, year	14.9 (2.6)	14.9 (2.5)	15.0 (2.9)	0.54
Systolic BP, mm Hg	144.4 (22.6)	141.8 (21.3)	147.6 (24.0)	0.14
Diastolic BP, mm Hg	86.4 (12.8)	85.4 (12.0)	87.0 (12.7)	0.71
MoCA score	21.9 (3.1)	21.2 (3.2)	23.4 (2.4)	0.003

*p-Value indicates the level of statistical significance of the comparisons between the African–Americans and the Caucasian–Americans using non-parametric Wilcoxon rank sum for age, education, blood pressure and MoCA, and chi-square test for sex.*

### MRI Acquisition

Magnetic resonance imaging data were acquired on a SIEMENS Trio 3-Tesla scanner available at Center for Systems Imaging of Emory University, Atlanta, GA, United States. Foam padding and ear forms were used to limit head motion and reduce scanner noise to the participants. High-resolution 3D anatomical images were acquired using sagittal T1-weighted magnetization-prepared rapid gradient echo with repetition time = 2300 ms, echo time = 2.89 ms, inversion time = 800 ms, flip angle = 8°, resolution = 256 × 256 matrix, slices = 176, thickness = 1 mm. The rsfMRI were collected axially for 170 volumes during 7.14 min by using an echo-planar imaging (EPI) sequence with repetition time = 2500 ms, echo time = 27 ms, flip angle = 90°, field of view = 22 cm, resolution = 74 × 74 matrix, slices = 48, thickness = 3 mm and bandwidth = 2598 Hz/pixel. We requested the participants to hold still, keep their eyes open and think nothing during the rsfMRI scan.

### Image Preprocessing and Time Series Extraction

**FIGURE 1:**
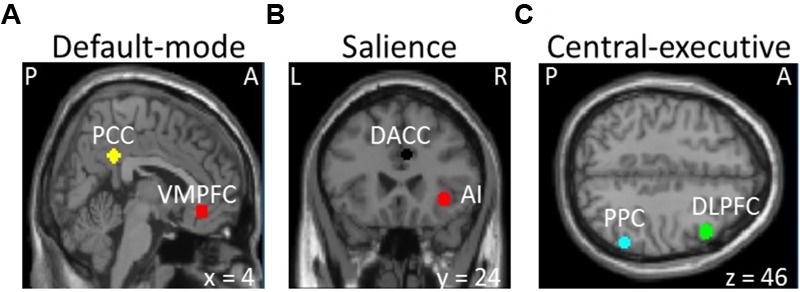
Selection of the **(A)** default-mode (VMPFC, ventromedial prefrontal cortex; PPC, posterior cingulate cortex), **(B)** salience (AI, insula; DACC, dorsal anterior cingulate cortex), and **(C)** central-executive (DLPFC, dorsolateral prefrontal cortex; PPC, posterior parietal cortex) networks (P, posterior; A, anterior; L, left; R, right).

Images were preprocessed for slice-timing correction, motion correction, co-registration to individual anatomical image, normalization to the Montreal Neurological Institute (MNI) template, and spatial smoothing of the normalized images with a 6 mm isotropic Gaussian kernel. The SPM12 (Wellcome Trust Centre for Neuroimaging, London, United Kingdom^1^) was used to perform those steps. We defined spherical regions of interest with 6 mm radius based on MNI coordinates centered at the posterior cingulate cortex (7, -43, 33) and ventromedial prefrontal cortex (2, 36, -10) of default-mode network, the insula (37, 25, -4) and dorsal anterior cingulate cortex (4, 30, 30) of salience network, and the posterior parietal cortex (54, -50, 50) and dorsolateral prefrontal cortex (45, 16, 45) of central-executive networks (see **Figure 1**) similar to the previous studies (Sridharan et al., 2008; Chand and Dhamala, 2016a). We selected the nodes only in the right hemisphere based on most prior neuroimaging studies that report the right-lateralized activations (Sridharan et al., 2008; Chen et al., 2013; Chand and Dhamala, 2016a). The MarsBaR software package^2^ was used to extract the voxel time courses of those nodes.

### Granger Causality Analysis

Multivariate analysis has become a commonplace to investigate the information flow between the brain areas and to study how such coordinated brain activity disrupts in diseases (Dhamala et al., 2008a,b; Chiong et al., 2013; Friston et al., 2013). Here we used Granger causality although other methods such as dynamic causal modeling, directed transfer function, and partial directed coherence provide the similar goals and results (Bajaj et al., 2016; Chand et al., 2016). The main benefits of using Granger causality are that it is a data-driven method and therefore computes the information flow based on the data itself at the nodes and networks level, relies on fewer assumptions about the underlying interactions, and does not need computationally intensive time/efforts as opposed to other methods such as dynamical causal modeling (Stephan et al., 2010; Chand and Dhamala, 2016b, 2017). Recent studies by our group and by other groups have successfully applied Granger causality to resting state and/or task fMRI data in both health and disease and have produced meaningful results in terms of information flow at the brain nodes and networks (Sridharan et al., 2008; Chiong et al., 2013; Liang et al., 2014; Bajaj et al., 2016; Chand and Dhamala, 2016a).

Granger causality can be mathematically expressed by considering simultaneously measured time series. Suppose we have two simultaneously recorded time series represented as, (1) X_1_(1), X_1_(2),..., X_1_(t),... and (2) X_2_(1), X_2_(2),..., X_2_(t). Granger causality analysis in the frequency (f) domain examines the strengths, directions, and frequencies of interactions between dynamic processes. Granger causality from the second time series ‘2’ to the first time series ‘1’ (i.e., from brain region ‘2’ to brain region ‘1’) is computed as (Dhamala et al., 2008a,b; Chand and Dhamala, 2016b),

(1) $$M_{2\to 1}(f)=-\ln(1-\frac{(\sum_{22}-\sum_{12}^{2}/\sum_{11})|H_{12}(f){|}^{2}}{S_{11}(f)})$$

where *H* is a transfer function, f represents a frequency-domain, S is spectral power, and ∑ is noise covariance. The value of Granger causality (*M*) varies between 0 and +∞, representing zero connectivity strength and maximum connectivity strength, respectively. If there are ‘*N*’ numbers of brain areas, the information outflow (*F*) at a node *i* can be calculated as,

(2) $$F_{i}=\frac{1}{N-1}\sum_{j}^{N}\left(M_{i\to j}-M_{j\to i}\right).$$

In our case, we have six nodes (two key nodes from each network). Therefore, an index *j* can be 1, 2, 3, 4, 5, and 6 nodes. The Granger causality outflow (also referred as the net information outflow) from the first node is *F*_1_ = [(*M*_1→2_ – *M*_2→1_) + (*M*_1→3_ – *M*_3→1_) + (*M*_1→4_ – *M*_4→1_) + (*M*_→5_ – *M*_5→1_) + (*M*_1→6_ – *M*_6→1_)]/5, where *M*_1→2_ is Granger causality from the first node to the second node, and *M*_2→1_ is Granger causality from the second node to the first node. Similarly, we calculate Granger causality outflows for other nodes. If the net outflow is negative at a node instead of the previously reported positive value in healthy individuals, then that node is said to have impaired directional connections.

### Cortical Thickness Calculation

FreeSurfer version 5.3^3^ was used to calculate the cortical thickness from T1-structural images. Briefly, this technique included spatial and intensity normalization, skull stripping, and an automated segmentation of cerebral white matter to locate the gray–white boundary (Dale et al., 1999). Cortical thickness was then computed from the distance between the gray–white boundary and the pial-surface (Fischl and Dale, 2000).

### Cognitive Test

We assessed the MoCA (Nasreddine et al., 2005) of each participant. The MoCA is a 30-point scale test administered in 10 min and assesses the global cognitive abilities. It encompasses the following sub-tests. The short-term memory recall (five points) consists of two learning trials of five nouns and delayed recall after 5 min. Visuospatial test includes a clock-drawing (three points) and a three-dimensional cube copy (one point). Executive functions test comprises the trail-making time B (one point), a phonemic fluency (one point), and a two-item verbal abstraction (two points). Attention, concentration, and working memory include a sustained attention (one point), a serial subtraction (three points), and digit forward (one point) and digit backward (one point). Language test consists of naming animals (three points), repetition of two syntactically complex sentences and fluency (two points). Orientation test comprises orientation to time and place (six points). Moreover, if participant’s formal education is 12 years or less, one point is added to his/her score. Previous studies (Nasreddine et al., 2005; Hachinski et al., 2006; Dong et al., 2010; Rossetti et al., 2011) suggest that the MoCA is more sensitive screening tool to define the MCI as compared with other existing screening tools such as mini-mental state examination (MMSE).

### Statistical Analysis

We first checked whether the data are normally distributed or not using Kolmogorov–Smirnov test. The age, education, MoCA, insular thickness and net flow were negatively skewed and the systolic and diastolic blood pressures were positively skewed (asymptotic *p*-value in the range 6.17 × 10^-71^ to 8.42 × 10^-14^) at the 0.05 significance level. As data variables did not show normal distributions before and after applying the appropriate transformations (square root, log, and reciprocal), we therefore chose the non-parametric alternative. We compared the sample characteristics between the African–Americans and the Caucasian–Americans using non-parametric Wilcoxon rank sum and/or chi-square test for discrete variables (e.g., sex). Net information outflows between the nodes of three networks were compared using non-parametric Wilcoxon rank sum test. The correlation analysis was performed using Spearman’s correlation. A *p*-value less than 0.05 was considered a statistically significant. MATLAB (Natick, MA, United States^4^) was used for analyzing the data.

## Results

### Interactions among the Salience, Central-Executive, and Default-Mode Nodes

**FIGURE 2:**
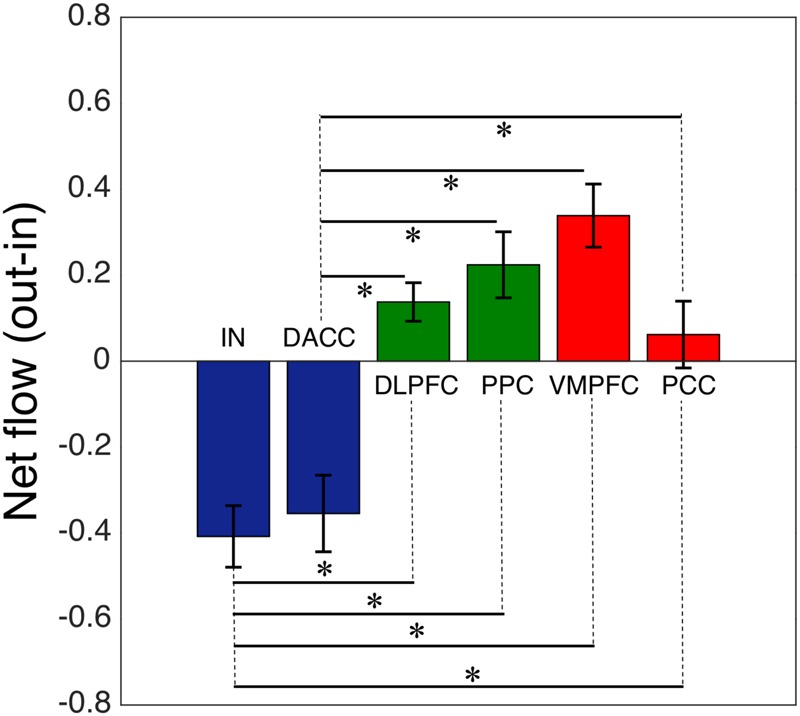
Net flow (information outflow minus information inflow) of the key nodes of the salience (IN, insula; DACC, dorsal anterior cingulate cortex), central-executive (DLPFC, dorsolateral prefrontal cortex; PPC, posterior parietal cortex) and default-mode (VMPFC, ventromedial prefrontal cortex; PPC, posterior cingulate cortex) networks. The AI and DACC of the salience network had a significantly lower net information flow compared with the central-executive and default-mode nodes (^∗^indicates statistical significance).

**FIGURE 3:**
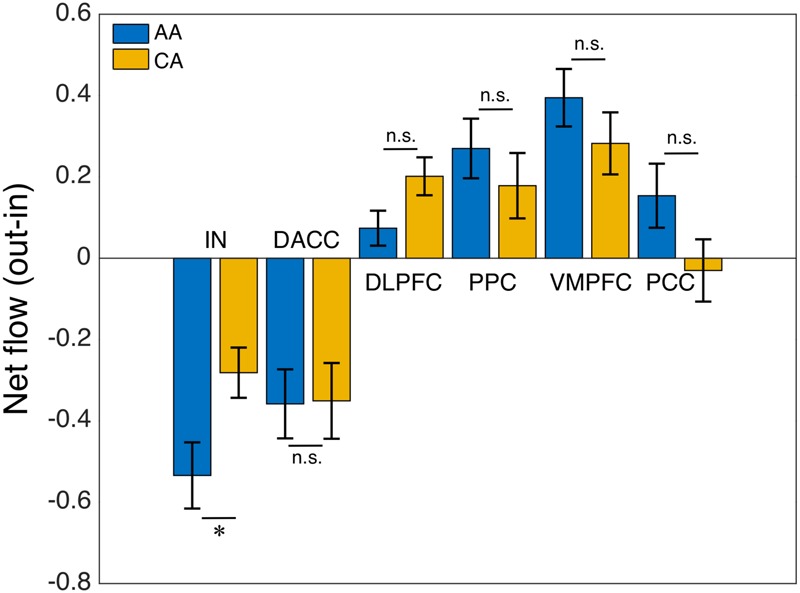
African–Americans (AA) versus Caucasian–Americans (CA) comparison: net flow (out-in) of the key nodes of the salience (IN, insula; DACC, dorsal anterior cingulate cortex), central-executive (DLPFC, dorsolateral prefrontal cortex; PPC, posterior parietal cortex) and default-mode (VMPFC, ventromedial prefrontal cortex; PPC, posterior cingulate cortex) networks. The AI of AA group had a significantly lower net flow (out-in) compared with that of CA group (^∗^indicates statistical significance; ns indicates not significant).

We computed the Granger causality between all possible pairs of the salience, central-executive, and default-mode nodes and calculated the net information outflow from each node. Our net outflow calculation showed that the nodes of salience network have significantly lower outflow than that of the central-executive and default-mode nodes (**Figure 2**) (Wilcoxon rank sum; *Z* = 10.52; *p* < 0.05). This negative information outflow of salience network nodes compared to previously reported positive flow in healthy individuals implied the impaired salience network nodes in our overall cohort. We compared the net information outflow between African Americans and Caucasian–Americans and found that the insula of salience network has significantly lower (negative value) in the African–Americans than in the Caucasian–Americans (Wilcoxon rank sum; *Z* = 2.06; *p* < 0.05) as shown in **Figure 3**.

### Cortical Thickness Comparison and Correlation

**FIGURE 4:**
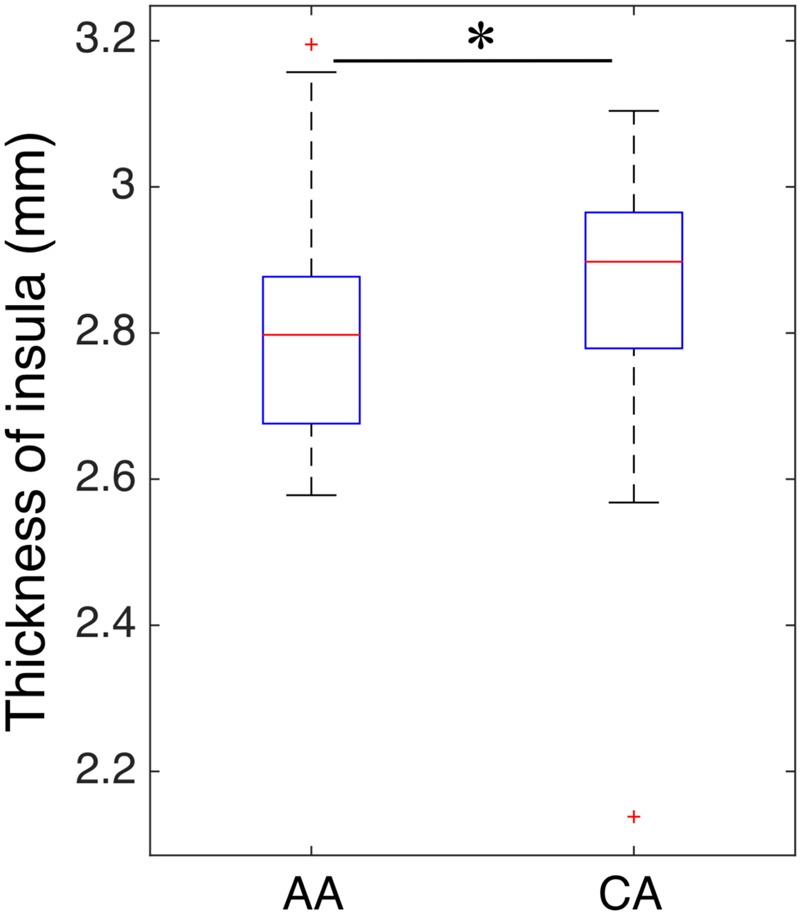
Comparison of insular thickness between African–Americans (AA) and Caucasian–Americans (CA). The thickness had a significantly lower value in AA group than that of CA group (^∗^indicates statistical significance).

**FIGURE 5:**
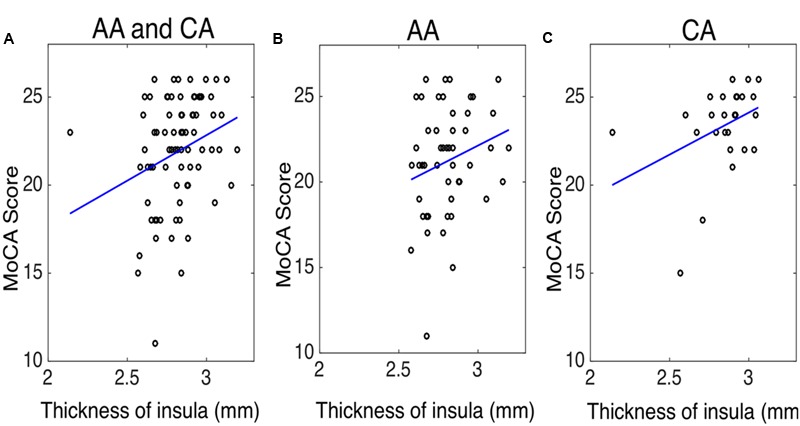
The correlation between the thickness of insula and the Montreal cognitive assessment (MoCA) score in **(A)** both African–Americans (AA) and Caucasian–Americans (CA) (*r* = 0.30 and *p* < 0.05), **(B)** AA (*r* = 0.19 and *p* = 0.18), and **(C)** CA (*r* = 0.40 and *p* < 0.05).

**FIGURE 6:**
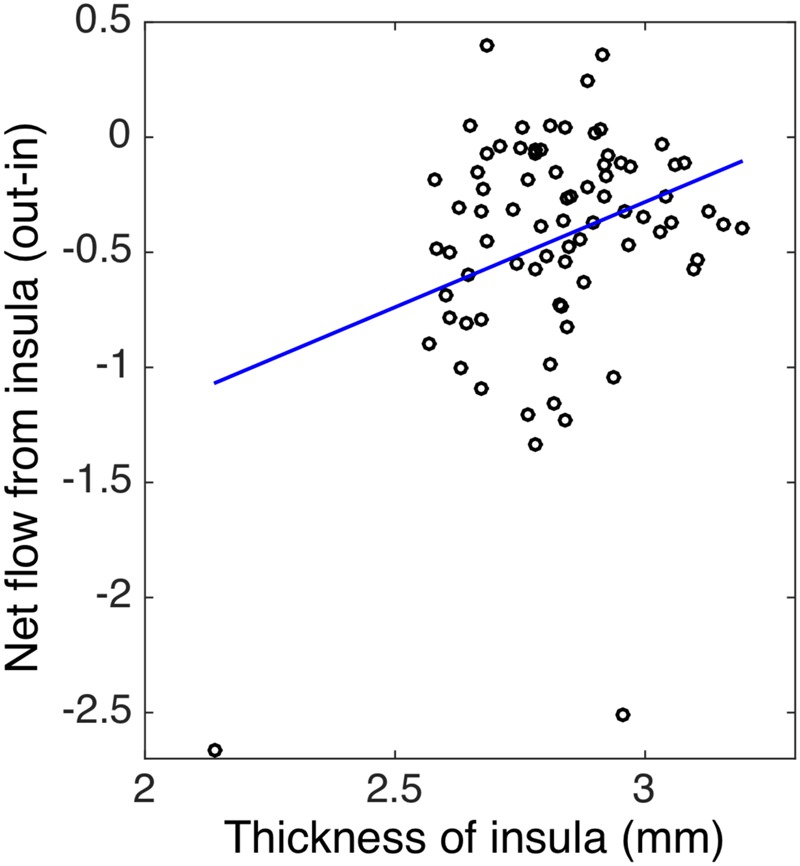
The correlation between the thickness and the net flow (out-in) of insula in both African–Americans (AA) and Caucasian–Americans (CA) (*r* = 0.31 and *p* < 0.05).

To evaluate whether structural alterations explain these connectivity differences, we measured the insular cortical thickness and compared the values between African–Americans and Caucasian–Americans as displayed in **Figure 4**. Cortical thickness in the African–Americans (median = 2.797 mm) and in the Caucasian–Americans (median = 2.897 mm) was significantly different (Wilcoxon rank sum; *Z* = 2.09; *p* < 0.05). To investigate how the thickness of impaired insular cortex relates with the performance on global cognitive testing, we performed a correlation analysis between the thickness and the MoCA scores. We found that a lower thickness was associated with lower performance reflected by MoCA scores (*r* = 0.30; *p* < 0.05). These results are provided in **Figure 5**. Furthermore, we found that the lower cortical thickness was correlated with the lower net information outflow at the insular cortex (*r* = 0.31; *p* < 0.05) as shown in **Figure 6**.

## Discussion

Here, we investigated the pattern of connectivity among the key brain areas of the salience, central-executive, and default-mode networks in hypertensive individuals with MCI and we compared African–Americans to Caucasian–Americans within this group. We found larger impairment in the control signal of the insula (of salience network) in the African Americans than in the Caucasian–Americans, as reflected by negative net information outflow metrics measured by Granger causality analysis. Although, there are very limited neuroimaging studies that investigate the neural bases of hypertension in cognitive decline (Li et al., 2015), our findings of greater information flow impairment in the African–Americans were in line with the previous non-neuroimaging reports that the African–Americans bear a greater risk of hypertension-associated cognitive impairment than the Caucasian–Americans (Redmond et al., 2011; Hajjar et al., 2016). We further examined the cortical thickness of impaired insula between the African–Americans and the Caucasian–Americans and found that the insula thickness of the African–Americans is significantly lower than that of the Caucasian–Americans. The insula thickness was found to be correlated with the behavior performance and with the net information flow at the insula cortex, respectively. Those results about insula thickness were also consistent with the existing literature that insula thickness decreases with cognitive decline (Hartikainen et al., 2012; Moretti, 2015), however the difference in insula thickness between the African–Americans and the Caucasian–Americans has not been previously explored.

Former studies consistently reported that the nodes of salience network render modulation effects over the default-mode and central-executive in healthy individuals (Sridharan et al., 2008; Goulden et al., 2014; Chand and Dhamala, 2016a). The controlling role of salience nodes over the other two networks has been argued to be structurally supported by direct white matter connections between the insula and the dorsal anterior cingulate cortex (Bonnelle et al., 2012; Jilka et al., 2014) and by their unique sharing of cytoarchitecture at neuronal level, i.e., only these regions consist of special type of neurons—von Economo neurons—that relay information processed within those nodes to other nodes, including the default-mode and executive nodes (Allman et al., 2005, 2010; Watson et al., 2006; Sridharan et al., 2008). Literature also shows that the insula of salience network is functionally connected to the central-executive network (Vincent et al., 2008), and has direct white matter connections to the other areas, including the inferior parietal lobe (Uddin et al., 2010), and temporo-parietal junction (Kucyi et al., 2012). These structural and functional settings show the great involvement of insula in many cognitive processes such as in the evaluation of task performance across varying perceptual and response demands (Uddin et al., 2010), the reorientation of attention in conscious error perception (or error awareness) (Ullsperger et al., 2010), and the switching between available cognitive resources to integrate external sensory information with internal states (Uddin and Menon, 2009). The dorsal anterior cingulate cortex of salience network is known for enhanced cognitive control (Egner, 2009) such as in switching activity in association with the insula during behaviorally harder tasks (Chand and Dhamala, 2016a). The above mentioned neural basis of control signal of the insula and the dorsal anterior cingulate cortex network (salience nodes) has been suggested to be crucial for cognitive maintenance in both task and resting states in cognitively healthy individuals, whereas impairment to such control activity might be caused by the underlying neuroanatomical changes, including the injuries to the highly sensitive/vulnerable von Economo neurons (Allman et al., 2005, 2010; Watson et al., 2006; Sridharan et al., 2008; Bonnelle et al., 2012).

Emerging evidence suggest atypical engagement of the insula of salience network in disease, including frontotemporal dementia, autism, schizophrenia, and Alzheimer’s disease (Menon, 2015; Uddin, 2015). The structural changes of the cortex, including the cortical thinning of insula with disease progression to MCI and/or Alzheimer’s disease, is widely reported in elderly people (Singh et al., 2006; Hartikainen et al., 2012; Moretti, 2015). Previous studies also suggested the link between the underlying structural changes and the corresponding functional changes of brain nodes and networks (Xie et al., 2012; Menon, 2015). Our connectivity findings indicated that the control mechanism of the salience network was impaired (negative value) in both the African–Americans and the Caucasian–Americans, and the insula was more impaired in the African–Americans than in the Caucasian–Americans. Although, the cortical thinning of insula has been consistently reported in cognitive impairment (Hartikainen et al., 2012; Moretti, 2015), there are no neuroimaging studies so far to our knowledge that report the racial disparity of insula thickness. Neuroimaging literature suggests that the insula and dorsal anterior cingulate cortex—the key regions of salience network—respond as the racially biased brain regions (Cao et al., 2015), especially the greater activity of insula to out-group race than in-group race (Lieberman et al., 2005; Liu et al., 2015), but there are no previous reports about the difference in connectivity patterns of those regions between the African–Americans and the Caucasian–Americans groups with hypertension and cognitive impairment. Prior non-neuroimaging studies repeatedly report that the African–Americans bear the greater risk of hypertension-associated cognitive impairment than the Caucasian–Americans (Redmond et al., 2011; Hajjar et al., 2016). Thus, our findings and existing neuroimaging/non-neuroimaging evidence taken together suggest that the insula is crucial in racial disparity in cognitively impaired individuals with hypertension. This study can be further extended in the future by including the groups of African–Americans and Caucasian–Americans with MCI but without a history of hypertension and by also including the cognitively healthy hypertensive and normotensive individuals to better dissociate the effect of hypertension alone in the brain nodes and networks.

In summary, we evaluated the patterns of interactions among the salience, default-mode, and central-executive nodes in the African–Americans and the Caucasian–Americans race groups, who had hypertension and cognitive impairment. We found that the insula of the salience network was functionally impaired greater, and had lower thickness in the African–Americans than in the Caucasian–Americans. Existing literature and our findings taken together thus suggest that the insula is potential biomarker in the cognitive disorders, including the racial disparity of cognitively impaired hypertensive population. It is worth nothing that the future research should direct toward dissociating the role of hypertension alone between the race groups in the insula and the other possible regions that are functionally and/or structurally connected to the insula.

## Author Contributions

Conceived and designed the experiment: IH, DQ. Performed the experiment: GC, JW, DQ, IH. Analyzed the data: GC, DQ, IH. Wrote the paper: GC, DQ, IH. Participated in the discussion and provided the comments: GC, JW, DQ, IH.

## Conflict of Interest Statement

The authors declare that the research was conducted in the absence of any commercial or financial relationships that could be construed as a potential conflict of interest. The reviewer AM and handling Editor declared their shared affiliation, and the handling Editor states that the process nevertheless met the standards of a fair and objective review.

## Footnotes

1 www.fil.ion.ucl.ac.uk/spm/software/spm12

2 http://marsbar.sourceforge.net

3 https://surfer.nmr.mgh.harvard.edu/

4 https://www.mathworks.com

## References

[B1] Allman J. M. Tetreault N. A. Hakeem A. Y. Manaye K. F. Semendeferi K. Erwin J. M. (2010). The von Economo neurons in frontoinsular and anterior cingulate cortex in great apes and humans. *Brain Struct. Funct.* 214 495–517. 10.1007/s00429-010-0254-0 20512377

[B2] Allman J. M. Watson K. K. Tetreault N. A. Hakeem A. Y. (2005). Intuition and autism: a possible role for Von Economo neurons. *Trends Cogn. Sci.* 9 367–373. 10.1016/j.tics.2005.06.008 16002323

[B3] Bajaj S. Adhikari B. M. Friston K. J. Dhamala M. (2016). Bridging the gap: dynamic causal modeling and Granger causality analysis of resting state fMRI. *Brain Connect.* 6 652–661. 10.1089/brain.2016.0422 27506256

[B4] Biswal B. Haughton V. M. Hyde J. (1995). Functional connectivity in the motor cortex resting human brain using echo-planar MRI. *Magn. Reson. Med.* 34 537–541. 10.1002/mrm.1910340409 8524021

[B5] Bonnelle V. Ham T. E. Leech R. Kinnunen K. M. Mehta M. A. Greenwood R. J. (2012). Salience network integrity predicts default mode network function after traumatic brain injury. *Proc. Natl. Acad. Sci. U.S.A.* 109 4690–4695. 10.1073/pnas.1113455109 22393019 PMC3311356

[B6] Bressler S. L. Menon V. (2010). Large-scale brain networks in cognition: emerging methods and principles. *Trends Cogn. Sci.* 14 277–290. 10.1016/j.tics.2010.04.004 20493761

[B7] Cao Y. Contreras-Huerta L. S. McFadyen J. Cunnington R. (2015). Racial bias in neural response to others’ pain is reduced with other-race contact. *Cortex* 70 68–78. 10.1016/j.cortex.2015.02.010 25798570

[B8] Chand G. B. Dhamala M. (2016a). Interactions among the brain default-mode, salience, and central-executive networks during perceptual decision-making of moving dots. *Brain Connect.* 6 249–254. 10.1089/brain.2015.0379 26694702

[B9] Chand G. B. Dhamala M. (2016b). The salience network dynamics in perceptual decision-making. *Neuroimage* 134 85–93. 10.1016/j.neuroimage.2016.04.018 27079535

[B10] Chand G. B. Dhamala M. (2017). Interactions between the anterior cingulate-insula network and the fronto-parietal network during perceptual decision-making. *Neuroimage* 152 381–389. 10.1016/j.neuroimage.2017.03.014 28284798

[B11] Chand G. B. Lamichhane B. Dhamala M. (2016). Face or house image perception: beta and gamma bands of oscillations in brain networks carry out decision-making. *Brain Connect.* 6 621–631. 10.1089/brain.2016.0421 27417452

[B12] Chao L. L. Pa J. Duarte A. Schuff N. Weiner M. W. Kramer J. H. (2009). Patterns of cerebral hypoperfusion in amnestic and dysexecutive MCI. *Alzheimer Dis. Assoc. Disord.* 23 245–252. 10.1097/WAD.0b013e318199ff46 19812467 PMC2760039

[B13] Chen A. C. Oathes D. J. Chang C. Bradley T. Zhou Z. W. Williams L. M. (2013). Causal interactions between fronto-parietal central executive and default-mode networks in humans. *Proc. Natl. Acad. Sci. U.S.A.* 110 19944–19949. 10.1073/pnas.1311772110 24248372 PMC3856839

[B14] Chiong W. Wilson S. M. D’Esposito M. Kayser A. S. Grossman S. N. Poorzand P. (2013). The salience network causally influences default mode network activity during moral reasoning. *Brain* 136(Pt 6) 1929–1941. 10.1093/brain/awt066 23576128 PMC3673466

[B15] Dale A. Fischl B. Sereno M. (1999). Cortical surface-based analysis. *Neuroimage* 9 179–194. 10.1006/nimg.1998.0395 9931268

[B16] Dhamala M. Rangarajan G. Ding M. (2008a). Analyzing information flow in brain networks with nonparametric Granger causality. *Neuroimage* 41 354–362. 10.1016/j.neuroimage.2008.02.020 18394927 PMC2685256

[B17] Dhamala M. Rangarajan G. Ding M. (2008b). Estimating granger causality from Fourier and wavelet transforms of time series data. *Phys. Rev. Lett.* 100:018701 10.1103/PhysRevLett.100.018701 18232831

[B18] Dong Y. Sharma V. K. Chan B. P. Venketasubramanian N. Teoh H. L. Seet R. C. (2010). The Montreal Cognitive Assessment (MoCA) is superior to the mini-mental state examination (MMSE) for the detection of vascular cognitive impairment after acute stroke. *J. Neurol. Sci.* 299 15–18. 10.1016/j.jns.2010.08.051 20889166

[B19] Egner T. (2009). Prefrontal cortex and cognitive control: motivating functional hierarchies. *Nat. Neurosci.* 12 821–822. 10.1038/nn0709-821 19554047

[B20] Elias M. F. Goodell A. L. Dore G. A. (2012). Hypertension and cognitive functioning: a perspective in historical context. *Hypertension* 60 260–268. 10.1161/HYPERTENSIONAHA.111.186429 22753214

[B21] Faraco G. Iadecola C. (2013). Hypertension: a harbinger of stroke and dementia. *Hypertension* 62 810–817. 10.1161/HYPERTENSIONAHA.113.01063 23980072 PMC3847558

[B22] Fischl B. Dale A. (2000). Measuring the thickness of the human cerebral cortex from magnetic resonance images. *Proc. Natl. Acad. Sci. U.S.A.* 97 11050–11055. 10.1073/pnas.200033797 10984517 PMC27146

[B23] Friston K. Moran R. Seth A. K. (2013). Analysing connectivity with Granger causality and dynamic causal modelling. *Curr. Opin. Neurobiol.* 23 172–178. 10.1016/j.conb.2012.11.010 23265964 PMC3925802

[B24] Gorelick P. B. Nyenhuis D. (2012). Blood pressure and treatment of persons with hypertension as it relates to cognitive outcomes including executive function. *J. Am. Hypertens.* 6 309–315. 10.1016/j.jash.2012.08.004 22995799

[B25] Goulden N. Khusnulina A. Davis N. J. Bracewell R. M. Bokde A. L. McNulty J. P. (2014). The salience network is responsible for switching between the default mode network and the central executive network: replication from DCM. *Neuroimage* 99 180–190. 10.1016/j.neuroimage.2014.05.052 24862074

[B26] Hachinski V. Iadecola C. Petersen R. C. Breteler M. M. Nyenhuis D. L. Black S. E. (2006). National Institute of Neurological Disorders and Stroke-Canadian Stroke Network vascular cognitive impairment harmonization standards. *Stroke* 37 2220–2241. 10.1161/01.STR.0000237236.88823.47 16917086

[B27] Hajjar I. Wharton W. Mack W. J. Levey A. I. Goldstein F. C. (2016). Racial disparity in cognitive and functional disability in hypertension and all-cause mortality. *Am. J. Hypertens.* 29 185–193. 10.1093/ajh/hpv084 26137951 PMC4989127

[B28] Hartikainen P. Rasanen J. Julkunen V. Niskanen E. Hallikainen M. Kivipelto M. (2012). Cortical thickness in frontotemporal dementia, mild cognitive impairment, and Alzheimer’s disease. *J. Alzheimers Dis.* 30 857–874. 10.3233/JAD-2012-112060 22466003

[B29] Henley N. M. (1969). A psychological study of the semantics of animal terms. *J. Verbal Learn. Verbal Behav.* 8 176–184. 10.1016/S0022-5371(69)80058-7

[B30] Humstone H. J. (1919). Memory span tests. *Psychol. Clin.* 12 196–200. PMC5076260 28909279

[B31] Jilka S. R. Scott G. Ham T. Pickering A. Bonnelle V. Braga R. M. (2014). Damage to the Salience Network and interactions with the Default Mode Network. *J. Neurosci.* 34 10798–10807. 10.1523/JNEUROSCI.0518-14.2014 25122883 PMC4131006

[B32] Johnson K. C. Margolis K. L. Espeland M. A. Colenda C. C. Fillit H. Manson J. E. (2008). A prospective study of the effect of hypertension and baseline blood pressure on cognitive decline and dementia in postmenopausal women: the Women’s Health Initiative Memory Study. *J. Am. Geriatr. Soc.* 56 1449–1458. 10.1111/j.1532-5415.2008.01806.x 18637980

[B33] Kimble M. O. Frueh B. C. Marks L. (2009). Does the modified Stroop effect exist in PTSD? Evidence from dissertation abstracts and the peer reviewed literature. *J. Anxiety Disord.* 23 650–655. 10.1016/j.janxdis.2009.02.002 19272751 PMC2844871

[B34] Kucyi A. Moayedi M. Weissman-Fogel I. Hodaie M. Davis K. D. (2012). Hemispheric asymmetry in white matter connectivity of the temporoparietal junction with the insula and prefrontal cortex. *PLoS ONE* 7:e35589 10.1371/journal.pone.0035589 PMC3334912 22536413

[B35] Li X. Liang Y. Chen Y. Zhang J. Wei D. Chen K. (2015). Disrupted frontoparietal network mediates white matter structure dysfunction associated with cognitive decline in hypertension patients. *J. Neurosci.* 35 10015–10024. 10.1523/JNEUROSCI.5113-14.2015 26157001 PMC6605417

[B36] Liang P. Li Z. Deshpande G. Wang Z. Hu X. Li K. (2014). Altered causal connectivity of resting state brain networks in amnesic MCI. *PLoS ONE* 9:e88476 10.1371/journal.pone.0088476 PMC3948954 24613934

[B37] Lieberman M. D. Hariri A. Jarcho J. M. Eisenberger N. I. Bookheimer S. Y. (2005). An fMRI investigation of race-related amygdala activity in African-American and Caucasian-American individuals. *Nat. Neurosci.* 8 720–722. 10.1038/nn1465 15880106

[B38] Liu Y. Lin W. Xu P. Zhang D. Luo Y. (2015). Neural basis of disgust perception in racial prejudice. *Hum. Brain Mapp.* 36 5275–5286. 10.1002/hbm.23010 26417673 PMC6868979

[B39] Menon V. (2011). Large-scale brain networks and psychopathology: a unifying triple network model. *Trends Cogn. Sci.* 15 483–506. 10.1016/j.tics.2011.08.003 21908230

[B40] Menon V. (2015). Salience Network. *Brain Mapp.* 2 597–611. 10.1016/b978-0-12-397025-1.00052-x

[B41] Moretti D. V. (2015). Conversion of mild cognitive impairment patients in Alzheimer’s disease: prognostic value of Alpha3/ Alpha 2 electroencephalographic rhythms power ratio. *Alzheimers Res. Ther.* 7:80 10.1186/s13195-015-0162-x PMC4696332 26715588

[B42] Nasreddine Z. S. Phillips N. A. Bedirian V. Charbonneanu S. Whitehead V. Collin I. (2005). The montreal cognitive assessment, MoCA: a brief screening tool for mild cognitive impairment. *J. Am. Geriatr. Soc.* 53 695–699. 10.1111/j.1532-5415.2005.53221.x 15817019

[B43] Novak V. Hajjar I. (2010). The relationship between blood pressure and cognitive function. *Nat. Rev. Cardiol.* 7 686–698. 10.1038/nrcardio.2010.161 20978471 PMC3328310

[B44] Oveisgharna S. Hachinski V. (2010). Hypertension, executive dysfunction, and progression to dementia. *Arch. Neurol.* 67 187–192. 10.1001/archneurol.2009.312 20142526

[B45] Pa J. Boxer A. Chao L. L. Gazzaley A. Freeman K. Kramer J. (2009). Clinical-neuroimaging characteristics of dysexecutive mild cognitive impairment. *Ann. Neurol.* 65 414–423. 10.1002/ana.21591 19399879 PMC2680500

[B46] Raichle M. E. (2015). The restless brain: how intrinsic activity organizes brain function. *Philos. Trans. R. Soc. Lond. B Biol. Sci.* 370 20140172 10.1098/rstb.2014.0172 PMC4387513 25823869

[B47] Redmond N. Baer H. J. Hicks L. S. (2011). Health behaviors and racial disparity in blood pressure control in the national health and nutrition examination survey. *Hypertension* 57 383–389. 10.1161/HYPERTENSIONAHA.110.161950 21300667 PMC3048351

[B48] Reitan R. M. (1958). Validity of the trail marking test as an indicator of organic brain damage. *Percept. Mot. Skills* 8 271–276. 10.2466/pms.1958.8.3.271

[B49] Rossetti H. C. Lacritz L. H. Cullum C. M. Weiner M. F. (2011). Normative data for the Montreal Cognitive Assessment (MoCA) in a population-based sample. *Neurology* 77 1272–1275. 10.1212/WNL.0b013e318230208a 21917776

[B50] Singh V. Chertkow H. Lerch J. P. Evans A. C. Dorr A. E. Kabani N. J. (2006). Spatial patterns of cortical thinning in mild cognitive impairment and Alzheimer’s disease. *Brain* 129(Pt 11) 2885–2893. 10.1093/brain/awl256 17008332

[B51] Sridharan D. Levitin D. J. Menon V. (2008). A critical role for the right fronto-insular cortex in switching between central-executive and default-mode networks. *Proc. Natl. Acad. Sci. U.S.A.* 105 12569–12574. 10.1073/pnas.0800005105 18723676 PMC2527952

[B52] Stephan K. E. Penny W. D. Moran R. J. den Ouden H. E. Daunizeau J. Friston K. J. (2010). Ten simple rules for dynamic causal modeling. *Neuroimage* 49 3099–3109. 10.1016/j.neuroimage.2009.11.015 19914382 PMC2825373

[B53] Stroop J. R. (1935). Studies of interference in serial verbal reactions. *J. Exp. Psychol.* 18 643–662. 10.1037/h0054651

[B54] Tombaugh T. N. (2004). Trail making test A and B: normative data stratified by age and education. *Arch. Clin. Neuropsychol.* 19 203–214. 10.1016/s08876177(03)00039-8 15010086

[B55] Troyer A. K. Moscovitch M. Winocur G. (1997). Clustering and switching as two components of verbal fluency: evidence from younger and older healthy adults. *Neuropshychology* 11 138–146. 10.1037/0894-4105.11.1.138 9055277

[B56] Uddin L. Q. (2015). Salience processing and insular cortical function and dysfunction. *Nat. Rev. Neurosci.* 16 55–61. 10.1038/nrn3857 25406711

[B57] Uddin L. Q. Menon V. (2009). The anterior insula in autism: under-connected and under-examined. *Neurosci. Biobehav. Rev.* 33 1198–1203. 10.1016/j.neubiorev.2009.06.002 19538989 PMC2743776

[B58] Uddin L. Q. Supekar K. Amin H. Rykhlevskaia E. Nguyen D. A. Greicius M. D. (2010). Dissociable connectivity within human angular gyrus and intraparietal sulcus: evidence from functional and structural connectivity. *Cereb. Cortex* 20 2636–2646. 10.1093/cercor/bhq011 20154013 PMC2951845

[B59] Ullsperger M. Harsay H. A. Wessel J. R. Ridderinkhof K. R. (2010). Conscious perception of errors and its relation to the anterior insula. *Brain Struct. Funct.* 214 629–643. 10.1007/s00429-010-0261-1 20512371 PMC2886909

[B60] Vicario A. Martinez C. D. Baretto D. Casale A. D. Nicolosi L. (2005). Hypertension and cognitive decline: impact on executive function. *J. Clin. Hypertens.* 7 598–604. 10.1111/j.1524-6175.2005.04498.x PMC8109513 16227762

[B61] Vincent J. L. Kahn I. Snyder A. Z. Raichle M. E. Buckner R. L. (2008). Evidence for a frontoparietal control system revealed by intrinsic functional connectivity. *J. Neurophysiol.* 100 3328–3342. 10.1152/jn.90355.2008 18799601 PMC2604839

[B62] Watson K. K. Jones T. K. Allman J. M. (2006). Dendritic architecture of the von Economo neurons. *Neuroscience* 141 1107–1112. 10.1016/j.neuroscience.2006.04.084 16797136

[B63] Xie C. Bai F. Yu H. Shi Y. Yuan Y. Chen G. (2012). Abnormal insula functional network is associated with episodic memory decline in amnestic mild cognitive impairment. *Neuroimage* 63 320–327. 10.1016/j.neuroimage.2012.06.062 22776459 PMC4513936

